# Discovery and Engineering of a Novel Bacterial L-Aspartate α-Decarboxylase for Efficient Bioconversion

**DOI:** 10.3390/foods12244423

**Published:** 2023-12-10

**Authors:** Wenjing Cui, Hao Liu, Yan Ye, Laichuang Han, Zhemin Zhou

**Affiliations:** School of Biotechnology, Jiangnan University, Wuxi 214122, China; wjcui@jiangnan.edu.cn (W.C.); 6200208115@stu.jiangnan.edu.cn (H.L.); 6220210035@stu.jiangnan.edu.cn (Y.Y.); zhmzhou@jiangnan.edu.cn (Z.Z.)

**Keywords:** panD, L-aspartate α-decarboxylase, β-alanine

## Abstract

L-aspartate α-decarboxylase (ADC) is a pyruvoyl-dependent decarboxylase that catalyzes the conversion of L-aspartate to β-alanine in the pantothenate pathway. The enzyme has been extensively used in the biosynthesis of β-alanine and D-pantothenic acid. However, the broad application of ADCs is hindered by low specific activity. To address this issue, we explored 412 sequences and discovered a novel ADC from *Corynebacterium jeikeium* (CjADC). CjADC exhibited specific activity of 10.7 U/mg and Km of 3.6 mM, which were better than the commonly used ADC from *Bacillus subtilis*. CjADC was then engineered leveraging structure-guided evolution and generated a mutant, C26V/I88M/Y90F/R3V. The specific activity of the mutant is 28.8 U/mg, which is the highest among the unknown ADCs. Furthermore, the mutant displayed lower Km than the wild-type enzyme. Moreover, we revealed that the introduced mutations increased the structural stability of the mutant by promoting the frequency of hydrogen-bond formation and creating a more hydrophobic region around the active center, thereby facilitating the binding of L-aspartate to the active center and stabilizing the substrate orientation. Finally, the whole-cell bioconversion showed that C26V/I88M/Y90F/R3V completely transformed 1-molar L-aspartate in 12 h and produced 88.6 g/L β-alanine. Our study not only identified a high-performance ADC but also established a research framework for rapidly screening novel enzymes using a protein database.

## 1. Introduction

L-aspartate α-decarboxylase (ADC, EC 4.1.1.11), a lyase that catalyzes L-aspartate to β-alanine and CO_2_, is an important metabolic enzyme involving the metabolic pathway of β-alanine and D-pantothenic acid [1,2,3,4]. In biotechnology, ADC has been widely applied to synthesize β-alanine and D-pantothenic acid via enzymatic transformation and metabolic engineering. β-alanine is the most promising C3 compound for producing diverse, high-value-added chemicals, such as D-pantothenic acid, carnosine, and acrylonitrile.

In bacteria, ADC is encoded by the panD gene, which is firstly translated to a precursor and then processed via self-cleavage, generally between G24 and S25, forming a mature enzyme harboring a pyruvoly group as a critical cofactor at the active center [5]. These pyruvoly-dependent ADCs are generally found in *Corynebacterium glutamicum* [6], *Bacillus subtilis* [7], and *Mycobacterium tuberculosis* [2]. Additionally, there are pyridoxal-5′-phosphate (PLP)-dependent ADCs found in insects like *Tribolium castaneum* [8,9] and *Aedes aegypti* [10]. The processes forming the active pyruvoly group of PanD precursor through post-translational modification can be categorized into two types: self-cleavage and activator-dependent cleavage. In self-cleavage, panD is firstly translated to an inactive pro-protein (π protein) and then undergoes a self process to form its own covalently bound prosthetic group, a pyruvoyl cofactor. This cofactor is created through intramolecular, non-hydrolytic serinolysis, where the side-chain oxygen of Ser25 attacks the carbonyl carbon of Gly24. The reaction, which is also known as a N→O acyl shift, results in the formation of an ester in processing [11]. The activator-dependent cleavage, on the other hand, involves a protein encoded by a separate gene, such as PanZ, catalyzing the cleavage between G24 and S25 [12]. 

ADCs from *C. glutamicum* and *B. subtilis* have been universally used in the biosynthesis of both β-alanine and D-pantothenic acid, either through enzymatic synthesis or metabolic engineering [13]. Recent studies have shown that ADC from *B. subtilis* (BsADC) has been more used in biosynthetic application because its specific activity was approximately higher than that of *C. glutamicum* and *T. castaneum* by two folds [14,15]. Additionally, ADC from *E. coli* was also investigated to produce β-alanine [3]. Although the ADCs from the sources above are extensively used in biosynthesis and bioconversion, their low activity still hinders the efficient preparation of these high-value-added products. Therefore, there is a high demand for the engineering and modification of these enzymes to improve the enzymatic activity and the maturation process to obtain more favorable ADCs for various applications.

Engineering ADCs typically focuses on two main aspects: enhancing enzymatic activity and improving the efficiency of self-processing. Many strategies for engineering ADCs are based on known crystal structures. Recently, a study on BsADC revealed that E56S mutation around the active center improved the enzymatic activity and catalytic stability. This improvement is likely associated with a decrease in catalytic inactivation [16]. Additionally, the K43Y mutation increased the specific activity by two times compared to the wild-type BsADC [17]. On the other hand, random mutagenesis on BsADC resulted in mutants V68I and I88M with slightly elevated specific activity. The mutation also marginally decreased the Km of the mutants compared with the wild-type enzyme [7]. More recently, a dual-mutant of BsADC, E56S/I88M, was used for the heterologous biosynthesis of β-alanine in *C. glutamicum*. This combinatorial mutant resulted in an increase in the overproduction of β-alanine but did not show a significant increase in specific activity [18].

Apart from enhancing enzymatic activity, several studies have focused on improving the efficiency of self-processing. Pyruvoyl-dependent enzymes, including several kinds of decarboxylases, undergo irreversible inactivation in the turnover condition due to a side reaction in the catalytic process, like the inactivation mechanism of the S-adenosylmethionine decarboxylase [19]. Although some critical residues, including V23, I26, T27, and E56, were implicated in restricting the autolytic self-processing of ADCs from different sources, no evolved mutants were obtained to improve the self-processing efficiency [20]. However, a recent study managed to engineer a mutant that relieved the mechanism-based inactivation by altering the protonation conformation of BsADC [21]. The limited number of ADCs with significantly improved enzymatic efficiency is due to high sequence similarity among different ADCs, insufficient genomic mining for discovering novel enzymes, and the scarcity of crystallographic structures in database. Hence, the discovery of novel ADCs with superior enzymatic properties and the evolution of wild-type enzymes to satisfy diverse application purposes are highly demanded.

In this study, we identified a novel and highly active ADC from *Corynebacterium jeikeium* (CjADC) by mining the Uniprot database with BLAST and Sequence Similarity Network (SSN) analysis. The enzymatic performance surpasses that of ADCs from *B. subtilis* and *C. glutamicum*. Leveraging structure-guided protein evolution, we successfully engineered the enzyme and revealed the proposed mechanism of evolution. The results not only provided a novel ADC for biotechnology applications but also shed light on a potential mechanism for evolving the enzymatic properties of ADC.

## 2. Materials and Methods

### 2.1. Strains and Culture Conditions

The strains used in this study are listed in Supplementary Table S1. *E. coli* JM109 and *E. coli* BL21(DE3) were used to propagate plasmid and overexpress the recombinant enzymes, respectively. All the strains were cultured in LB medium with 100 μg/mL of Ampicillin or 50 μg/mL of Kanamycin.

### 2.2. Plasmid Construction and Gene Mutagenesis

Different sources of L-aspartate α-decarboxylase-encoding panD genes were synthesized and cloned into pET28a with His-Tag fused to the C-termini. Then, the recombinant plasmids were transformed into *E. coli* JM109 for propagation. After the recombinant plasmid from *E. coli* JM109 was prepared, the plasmids were then transformed into *E. coli* BL21(DE3) for overexpression of panD genes from different sources and the corresponding mutants. The primers used for cloning and mutagenesis used in this study are shown in Supplementary Table S2.

Mutagenesis of panD gene from *C. jeikeium* was performed through reverse PCR using the plasmid pET-28a-CorJei as a template, and the primers used for this are shown in Table S2. Then, DpnI was added to the PCR product and digested at 42 °C for 1 h to eliminate the template plasmid. Accordingly, the digested products were purified using the fast DNA product purification kit.

### 2.3. Expression and Purification of Recombinant Enzymes

The *E. coil* BL21 (DE3) harboring the panD gene from different sources and the mutants were inoculated in 5 mL LB medium with kanamycin and cultured in a shaker of 37 °C and 200 rpm for 12 h. Then, 1% (*v*/*v*) of the culture was inoculated into LB medium containing 50 μg/mL kanamycin and cultured at 37 °C with shaking of 200 rpm. When the culture reached a cell density of OD600 = 0.6–0.8, 0.3 mM of IPTG was added to induce the expression of recombinant enzymes at 25 °C for about 16–18 h.

For the purification of L-aspartate α-decarboxylase, after induction, the cell cultures were collected and re-suspended in the binding buffer containing of 20 mM imidazole dissolved in phosphate (pH 6.5). The cell suspension was then subjected to ultrasonication on ice. After ultrasonication, centrifugation was carried out to separate the soluble and insoluble fractions. The supernatants were then collected for the next purification. The enzyme was purified using a HisTrap FF column after filtration through a 0.22 μm film. The purified enzymes were dialyzed at 4 °C for 8 to 12 h in 50 mM of phosphate buffer at pH 6.5, and the dialysate was changed twice during dialysis. The purity of the purified enzyme was detected using SDS-PAGE. The concentration of the purified enzyme was determined by measuring the absorbance at λ280 and then calculated using the webtool Protein Concentration Calculator (https://www.aatbio.com/tools/calculate-protein-concentration, 5 May 2022).

### 2.4. Structure Modeling and Molecular Docking

The protein structures of wild-type CjADC and the mutant of octamer CjADC were built using the AlphaFold2.3.1 with the AlphaFold-Multimer mode [22]. The structure of substrate L-Asp was obtained from PubChem (https://pubchem.ncbi.nlm.nih.gov/, 16 November 2022) and was docked into the catalytic pocket of CjADC using the Glide dock of Schrodinger 2021-4 with the standard precision mode. Each molecular docking generates 20 poses, and the best pose is selected through scoring ranking and expert judgment. The structure of the enzyme–substrate complex was further optimized using the relax module of the Rosetta 2021.16.61629 bundle [23]. The non-bonding interactions were analyzed using the web tool PLIP (https://plip-tool.biotec.tu-dresden.de/plip-web/plip/index, 4 December 2022).

### 2.5. Mutagenesis and Screening of L-Aspartate α-Decarboxylase

The amino acid sites in the range of Rosetta_ddg substrate active pocket 5 Å were used for virtual saturation mutation, and the change in unfolding free energy before and after mutation was obtained with the aid of calculation. According to the results of conservative analysis, after excluding the associated mutations of ultra-conservative sites, the sites with significantly reduced binding free energy were selected as mutation hotspots, and degenerate primers were designed to saturate mutation hotspots.

Through the modified Combinatorial Active-site Saturation Test (CAST) method, the selected sites were mutated using saturation mutation using NNK degenerate primers. After reverse PCR, *E. coli* BL21 (DE3) was transformed, and the single colonies were selected from the plates and then inoculated into a 96-well plate. After 16 h induction, the plates were centrifuged, and the supernatants were discarded. Then, L-aspartate sodium with a final concentration of 500 mM was added to the plates and incubated at 37 °C for 30 min. Afterward, the reactants were centrifuged, and the supernatants were then placed on the thin plate and screened using thin-layer chromatography. The mutants with higher levels of β-alanine were selected, and the mutated sequences were determined through sequencing.

### 2.6. Measurement of Specific Activity

The specificity activity of the wild-type L-aspartate α-decarboxylase from different sources and the mutants was determined through the measurement of the amounts of the products using high-performance liquid chromatography (HPLC). The total volume of the enzymatic reaction solution was 1 mL, and the L-aspartate stock solution was diluted with phosphate buffer at pH 7.0, and the final concentration was 100 mM. After preheating for 10 min at 37 °C, 0.1 mg of pure enzyme was added to the reaction solution. After 10 min, the reaction was inactivated using 100 °C incubation treatment for 15 min. The amount of product was measured using HPLC analysis. One unit is defined as the amount of the enzyme that catalyzes the conversion of one micromole of L-aspartate per minute under the specified conditions. The experiments were independently replicated in triplicate. The data are shown as mean ± s.d.

### 2.7. Determination of Kinetic Parameters

The final concentration of sodium L-aspartate (1, 2, 5, 10, 20, 50, 80, 100 mM) was added into the pure enzyme solution (final concentration is 0.1 mg/mL) and was incubated at 37 °C and pH 7.0 for 5 min, and then the reaction solution was heated at 100 °C and 15 min to terminate the reaction. The concentration of the product β-alanine was measured using HPLC according to the above procedure. The function of Michaelis–Menten provided by GraphPad Prism9 software (Ver. 9.0) was applied to calculate the kinetic parameters. The experiments were independently replicated in triplicate. The data are shown as mean ± s.d.

### 2.8. Characterization of Optimum Temperature and Themostability

The pure recombinant ADC (0.1 mg) and the substrate solution were preheated at 30 °C, 37 °C, 40 °C, 45 °C, 55 °C, 60 °C, 65 °C, 70 °C, and 80 °C for 5 min. Then, 100 mM L-aspartate (final concentration) was added into the reaction system with a pH of 7.0. After reaction, the enzyme activity was determined, and the optimal reaction temperature of the enzyme was analyzed. The experiments were independently replicated in triplicates. The data are shown as mean ± s.d.

To determine the thermostability, 0.1 mg of recombinant ADC solution was incubated at 70 °C at different times and then cooled on ice for 5 min. The final concentration of 100 mM L-aspartate was added into the reaction system. The reaction was performed at optimal temperature and pH 7.0 for 10 min. The maximal enzyme activity was defined as 100%. Then, the residual enzyme activity was determined. The experiments were independently replicated in triplicate. The data are shown as mean ± s.d.

### 2.9. Characterization of the Optimal pH and pH Stability

To determine the optimal pH, 0.1 mg of recombinant ADC was preheated for 5 min at 37 °C and then transferred to a reaction solution (50 mM Na_2_HPO_4_/NaH_2_PO_4_) containing 100 mM sodium L-aspartate at pH 3.0, 4.0, 4.5, 5.0, 5.5, 6.0, 7.0, 7.5, 8.0, and 9.0. After 10 min of reaction, the enzyme activity was determined according to the above procedure, and the optimum reaction pH of the enzyme was analyzed. The experiments were independently replicated in triplicate. The data were shown by mean ± s.d.

To determine the pH stability, 0.1 mg of the purified wild-type CjADC and the mutant were dissolved in the same buffer with different pH (pH 3.0, 4.0, 5.0, 6.0, 7.0, 8.0, 9.0), then incubated for 5 h. Afterwards, the ADC solution was added into 100 mM L-sodium aspartate solution (pH 7.0). Then, the reaction solution was incubated at 37 °C for 10 min. When the reaction is completed, the residual enzyme activity was determined according to the above procedure. The maximal enzyme activity was defined as 100%, and the effect of pH on enzyme stability was analyzed. The experiments were independently replicated in triplicates. The data are shown as mean ± s.d.

### 2.10. Determination of Melting Temperature (Tm)

The Tm values of CjADC and the mutant were determined using Nano DSC (Waters^TM^ TA instruments, New Castle, DE, USA). The ultra-pure water and dialysate were degassed, the reference pool and sample pool were first moistened with ultra-pure water, and then the dialysate was moistened and filled with the reference pool and sample pool. The temperature scanning interval was set to 30~130 °C, the pressure was 3 atm, and the heating rate was 1 °C per minute. Tm values were obtained by processing the data using the Nano built-in analysis software (DSCRun v4.8.9) and fitting them using the Two-State Scaled model. The experiments were independently repeated in triplicate.

### 2.11. Molecular Dynamics (MD) Simulation and Structural Analysis

All MD simulations in this study were carried out with NAMD 2.14 using CHARMM36m force field [24,25]. The input files for MD simulation were generated with the assistance of the web tool CHARMM-GUI (https://charmm-gui.org/, version 3.7, 12 December 2022) [26,27], and the CHARMM topology and parameter files for the substrate-free L-Asp were generated using CGenFF [28]. In the present study, the acetylation-modified N terminus was used to mimic the pyruvoyl group in the active site since this force field of pyruvoyl-modified residue is not available in the current CHARMM force field. The enzyme–substrate complex was solved in a TIP3P explicit solvent box using 10 Å as the edge distance, and the system was neutralized using 0.15 M K+ and Cl-. In MD simulation, the time step was 2 fs, the cutoff for non-bonded interactions was 12 Å, and the periodic boundary conditions and particle mesh Ewald were applied. The MD simulation was carried out in three steps, including 1 ns of equilibration in NVT ensemble under 300 K, heating from 0 K to under 300 K with a gradient of 5 K and 50 ns of production MD simulation in the NPT ensemble under 300 K. The RMSD and RMSF were analyzed using Bio3D [29].

### 2.12. Fed-Batch Cultivation in 5 L Fermenter

Fed-batch cultivations were carried out in a 5 L bioreactor based on a previously published method with slight modification [30]. In brief, recombinant *E. coli* cells harboring CjADC and the mutant were inoculated at a volume ratio of 6% (*v*/*v*) into a 5 L bioreactor containing 3 L of medium. The initial pH of the fermenter system was adjusted to 7.0, the initial culture temperature was set at 37 °C, and the initial rotation speed was set to 200 rpm. After 6 h of fermentation, exponential feeding was initiated to maintain the specific growth rate of the bacteria at about *μ* = 0.2 h, while the pH was coupled to Dissolved Oxygen (DO) and maintained at 7.0. The exponential fed feeding approach utilized the following formula, where *F*(*t*) represents the feeding rate:

$$F\left(t\right)=\frac{0.2X_{0}V_{0}{exp}^{{(\mu }_{set}t)}}{0.46(S_{f}-S_{0})}$$

Here, *X*_0_ refers to the dry weight of cells in the initial stage of feeding (L/h), *V*_0_ represents the initial medium volume of the medium (L), *t* represents the feeding time (h), and *S_f_* and *S*_0_ denote the glucose concentrations in the feeding medium and the fermentation broth after each sampling, respectively (g/L).

The samples were collected every 2 h to measure the optical density at 600 nm wavelength (OD600) and the remaining glucose concentration. When the OD600 of the bacteria biomass reached approximately 60, we adjusted the temperature in the fermentation tank to 30 °C, and 0.4 mM IPTG was added. IPTG injection was conducted every two hours, and samples were taken at the same time intervals to monitor the biomass OD600 of the bacteria biomass. The fermentation terminated after 30 to 32 h of cultivation.

### 2.13. Whole-Cell Bioconversion

The successfully expressed strain was induced by shaking the bottle, and the recombinant strain with OD600 = 40 was collected for reaction in a 10 mL system. In the 10 mL reaction system, the concentration of substrate L-aspartate was 1 M, which was dissolved in the 20 mM phosphate-buffered saline (pH 7.0). The first sampling was performed after 2 h of bioconversion. The samples were then taken at regular intervals to measure changes in the substrate and product via HPLC.

## 3. Results and Discussion

### 3.1. Mining a Novel L-Aspartate α-Decarboxylase

**Figure 1:**
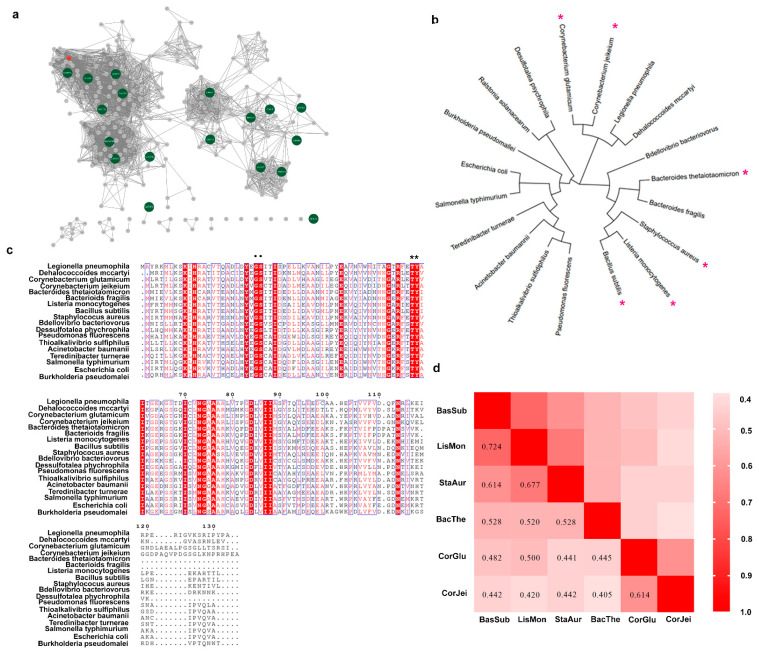
The procedure for mining of novel L-aspartate α-decarboxylase employing SSN analysis and sequence alignment. (**a**) Sequence similarity network of L-aspartate α-decarboxylase retrieved from UniProtKB in precision mode. The green nodes stand for the selected sequence for the following analysis. (**b**) Phylogenetic analysis of L-aspartate α-decarboxylase from eighteen sources. The red asterisks represent the sources for the next pairwise alignment. (**c**) Multiple-sequence alignment of diverse L-aspartate α-decarboxylase. Highly conserved residues are denoted in red boxes, and the residues associated with catalysis are denoted by black dots and asterisks. (**d**) Pairwise alignment of L-aspartate α-decarboxylase from eight sources. BacSub stands for enzymes from *Bacillus subtilis*. Similarly, LisMon stands for *Listeria Monocytogenes*; StaAur stands for *Staphylococcus aureus*; BacThe stands for *Bacteroides thetaiotaomicron*; CorGlu stands for *Corynebacterium glutamicum*; CorJei stands for *Corynebacterium jeikeium*. The numbers in the boxes represent the sequence homology of L-aspartate α-decarboxylase between the two sequences.

To discover novel ADCs, a BLAST search across Uniprot database was first performed using L-aspartate α-decarboxylase from *C. glutamicum* as a probe. Then, 412 candidate sequences were retrieved from the database. To further analyze these sequences, the EFI-EST web tool was employed to generate a sequence similarity network (SSN) [31,32]. The sequences on the SSN were classified into six distinct clusters. We selected nineteen sequences from these clusters, and the sequence identity was higher than 40% in the sequence alignment. We then used these sequences to construct a phylogenetic tree using the maximum-likelihood method in MEGA 7 software (ver. 7) (Figure 1b). Additionally, we performed a protein sequence alignment to further analyze the sequence data (Figure 1c). The results revealed that the L-aspartate α-decarboxylase probe, encoded by the panD gene of *C. glutamicum*, was in a different evolutionary lineage from that of *B. subtilis*, despite both enzymes belonging to the pyruvoyl-dependent enzyme class. However, both enzymes shared the G24-S25 conserved motif, involved in cleaving [1,11], and T57-Y58, involved in catalysis [11,33]. Given that the ADCs from these two sources displayed different enzymatic activities, we speculated that the enzymatic properties were not closely related to the evolutionary relationship shown on the phylogenetic tree. Therefore, due to their superior enzymatic activity, we selected six sequences, including those from both *C. glutamicum* and *B. subtilis*, for further characterization. We compared the sequence conservation using pairwise alignment and found that these sequences exhibited high or medium conservation. Subsequently, we chose four typical sequences from different lineages related to *B. subtilis* and *C. glutamicum* for additional characterization.

### 3.2. Comparison of the Whole-Cell Transformation Capacity with Different ADCs

**Figure 2:**
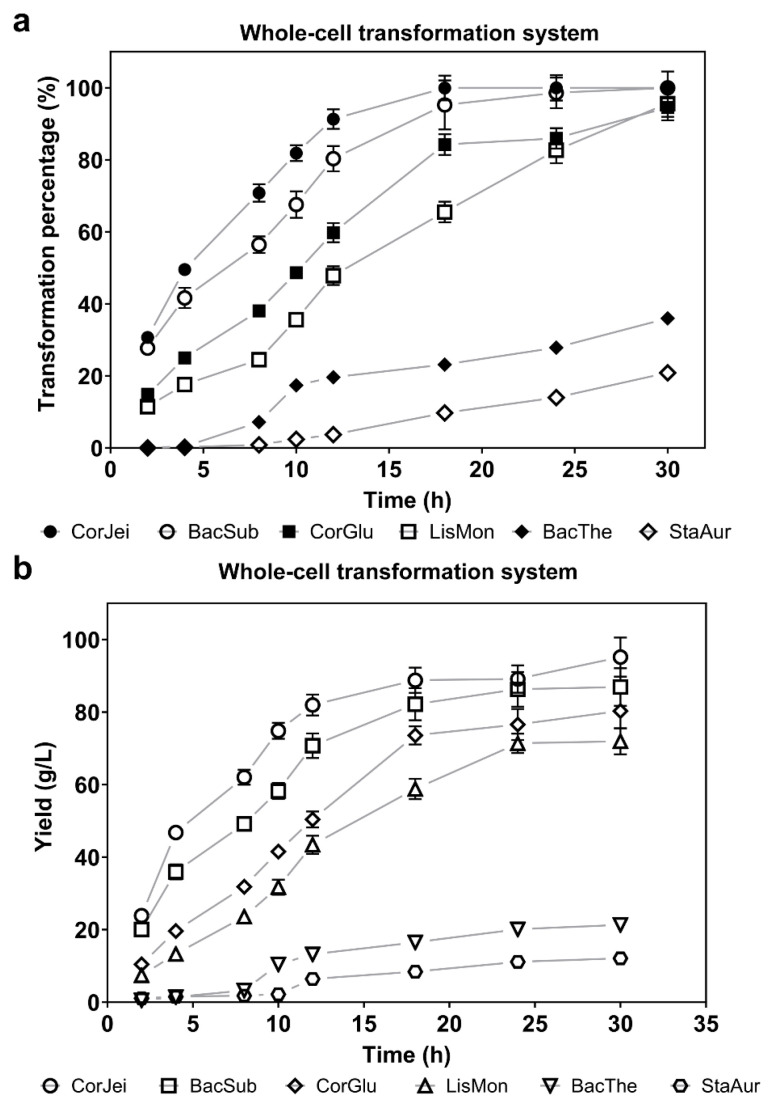
Determination and comparison of the transformation efficiency of β-alanine using a whole-cell bioconversion system with L-aspartate α-decarboxylase. (**a**) The transformation efficiency of L-aspartate using whole-cell bioconversion systems harboring L-aspartate α-decarboxylase from six sources over the transformation time. Sodium L-aspartate (1 M) was used as the substrate in the whole-cell systems. The cell density in each system was adjusted to OD600 = 40. (**b**) The yield of β-alanine produced by L-aspartate α-decarboxylase from the six sources is depicted over time. The concentration of substrate and the cell density used in the whole-cell systems were the same as in (**a**). All the experiments were independently repeated in triplicate. The data are shown as mean ± s.d.

To compare the catalytic capacity of the six ADCs, we initially utilized a whole-cell system to evaluate the percentage of substrate transformation and the yields of the produced compound mediated by recombinant cells containing panD genes. Following induction, the recombinant cells were cultured to a density (OD600) of approximately 40 in a 5 mL system. Subsequently, a substrate solution of 1 M L-sodium aspartate was added to initiate the transformation process. The first sample was collected after 2 h, and subsequent samples were taken at regular intervals. By evaluating the level of β-alanine, we found that ADC derived from *Corynebacterium jeikeium* (CjADC) converted approximately 50% of the substrate after 10 h transformation, exhibiting the highest catalytic efficiency and completely converting the 1 M substrate after 28 h (Figure 2a). The final yield of β-alanine transformed by CjADC was 85.05 g/L, which was higher than that of the *B. subtilis* ADC (Figure 2b). The *B. subtilis* ADC, which has generally been used for the overproduction of β-alanine in various hosts due to its higher enzymatic activity compared to that of *C. glutamicum*, completely converted the 1 M substrate after 32 h of bioconversion. The conversion time was longer than CjADC. These data indicate that CjADC is a potential enzyme that outperforms ADC from *B. subtilis* (BsADC) that has been widely used to synthesize β-alanine through metabolic engineering [18,34,35].

### 3.3. Determination of the Enzymatic Properties

**Figure 3:**
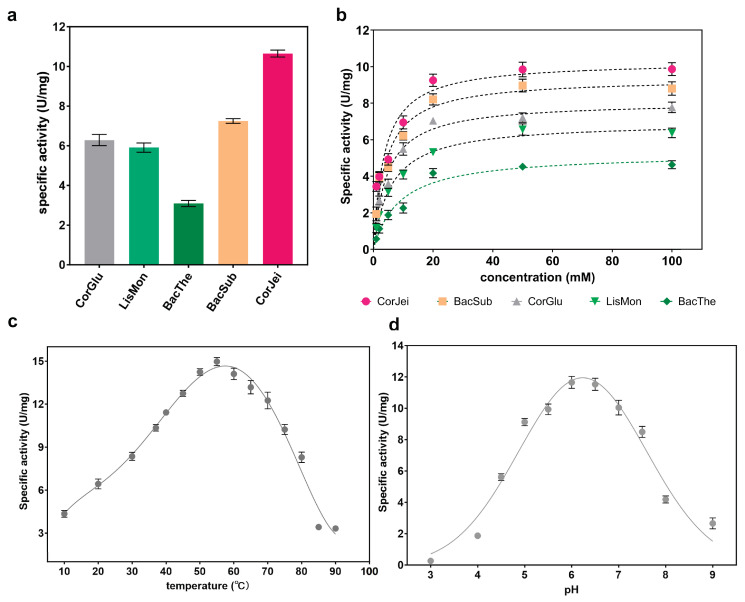
Determination and characterization of the enzymatic properties of L-aspartate α-decarboxylase from different sources. (**a**) Determination and comparison of the specificity of L-aspartate α-decarboxylase from different sources. The abbreviated enzymes are the same as those mentioned in Figure 1. (**b**) The specific activity of L-aspartate α-decarboxylase from six sources against the concentration of substrate. (**c**) Determination of the optimal temperature of L-aspartate α-decarboxylase from *C. jeikeium* (CjADC). (**d**) Determination of the optimal pH of L-aspartate α-decarboxylase from *C. jeikeium* (CjADC). All the experiments were independently repeated in triplicate. The data were shown in mean ± s.d.

To further compare the enzymatic properties of these ADCs, we expressed panD genes from different sources in *E. coli* BL21(DE3) cells. Among them, five of the ADCs were successfully expressed as soluble proteins, while panD from *Staphylococcus aureus* was produced as inclusion bodies. The five ADCs were then purified and verified using SDS-PAGE (Supplementary Figure S1). The specific activities of these ADCs were determined. The results showed that CjADC exhibited the highest specific activity of 10.7 U/mg, which was 1.5 times higher than 7.2 U/mg of BsADC (Figure 3a). Furthermore, the kinetic parameters Km and Kcat were determined (Figure 3b), and we observed that CjADC displayed the lowest Km and highest Kcat, as well as Kcat/Km, among the five ADCs, indicating that CjADC has a higher catalytic efficiency. In addition, the Kcat and Kcat/Km of CjADC were 1.17 and 1.3 times higher, respectively, than that of BsADC (Table 1). These results suggest that CjADC has higher catalytic performance than BsADC.

Next, to comprehensively characterize the enzymatic properties, we determined the optimal temperature and pH of CjADC. Through Gaussian regression analysis, we found that the optimal temperature for CjADC was 53.5 °C, and the optimal pH was 6.2 (Figure 3c,d). Overall, these findings provide a comprehensive characterization of the enzymatic properties of CjADC and highlight its potential as a highly efficient enzyme in various applications.

### 3.4. Generation of Single-Site Mutations and Screening of the Mutants

Although CjADC shows the highest enzymatic activity, the specific activity and other enzymatic parameters are not significantly higher than those commonly used [4,36,37]. Thus, evolving CjADC for improved enzymatic properties is in high demand. Because there are no high-throughput methods for ADCs, we employed a structure-guided semi-rational design for this purpose.

**Figure 4:**
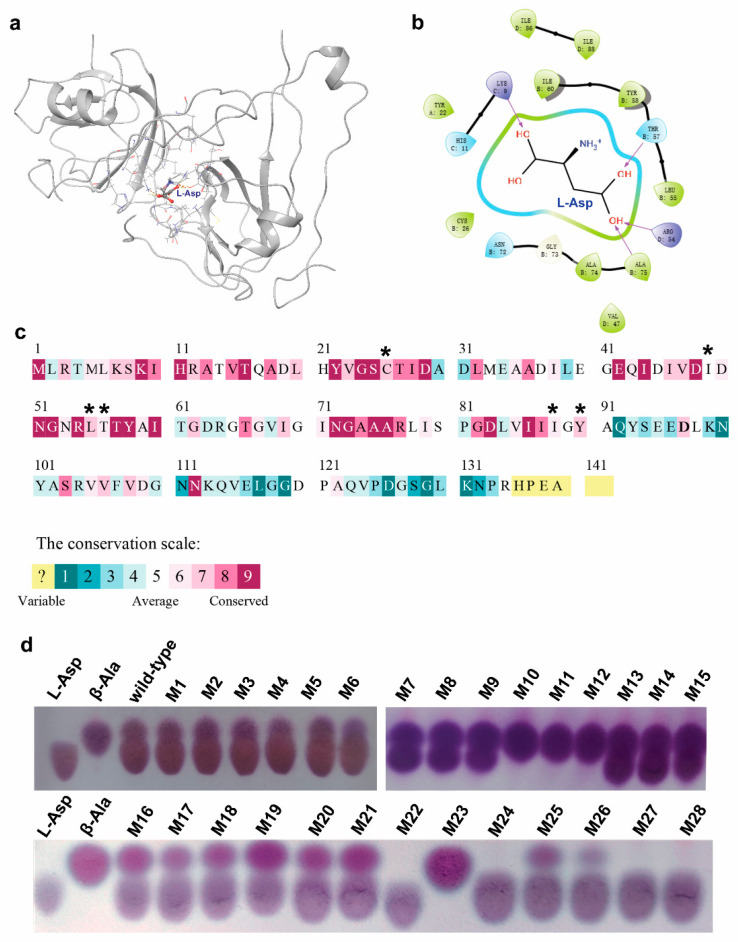
Mutagenesis of CjADC based on the structure analysis. (**a**) Overview of the structure of L-aspartate-bounded single unit of CjADC. L-Asp stands for the substrate L-aspartate and is shown in a stick-and-ball structure. The residues 5 Å around L-aspartate are shown in a stick structure. (**b**) The simplified diagram shows the residues associated with L-aspartate. The purple arrows point out the predicted interaction between groups of L-aspartate and the specific residues. (**c**) The analysis of L-aspartate α-decarboxylase based on multiple-sequence alignment. The asterisks denoted average and medium conserved residues. (**d**) Thin-layer chromatography analysis of the mutants obtained from the mutagenesis libraries of CjADC transforming L-aspartate to β-alanine. The mutants were cultured and induced using IPTG for 16 h. Then, 500 mM of L-aspartate was added and incubated for 30 min at 37 °C. After induction, 5 μL of the samples and 100 mM of the standards were dotted on the plates.

To this end, we firstly predicted the quaternary structure of CjADC using AlphaFold2 software (AF2). The predicted structure was then analyzed using PROCHECK to assess the overall model geometry, including residue-by-residue geometry (Supplementary Figure S2). More than 92% of residues were found to be distributed in the most favored region (Supplementary Figure S2a, the red region), indicating the suitability of the model for substrate docking analysis. Subsequently, the substrate L-aspartate was docked into the predicted structure model of CjADC to form an enzyme–substrate complex structure (Figure 4a).

To identify the potential residues involved in substrate binding and catalysis, the residues within 5 Ångstroms (Å) of L-aspartate were analyzed (Figure 4b). Within this region, 22 residues were identified, showing varying degrees of conservation (Figure 4c). Highly conserved residues, such as R54, T57, and Y58, were excluded due to their importance in catalysis [1,11,33]. Finally, eight residues (H11, C26, I49, L55, T56, N72, I88, Y90) were chosen using Rosetta_ddg for virtual mutagenesis analysis. The mutation results were sorted based on the binding free energy between CjADC and L-aspartate. The residues C26, I49, L55, T56, I88, and Y90 exhibited significant decreases in binding free energy after mutation (Figure 4c and Supplementary Table S3). Thus, they were selected for further saturation mutation. Oligos with degenerate codons NNK were used to generate mutagenesis libraries for each of these mutation sites. Among the approximately 1036 colonies screened from all six libraries, 28 showed detectable β-alanine production in the whole-cell bioconversion system, with 4 colonies (M10, M11, M12, and M23) displaying a high transformation level, completely converting L-aspartate to β-alanine (Figure 4d). After sequencing, the mutations were identified as I88L, Y90F, C26V, and I88M (Table 2).

**Figure 5:**
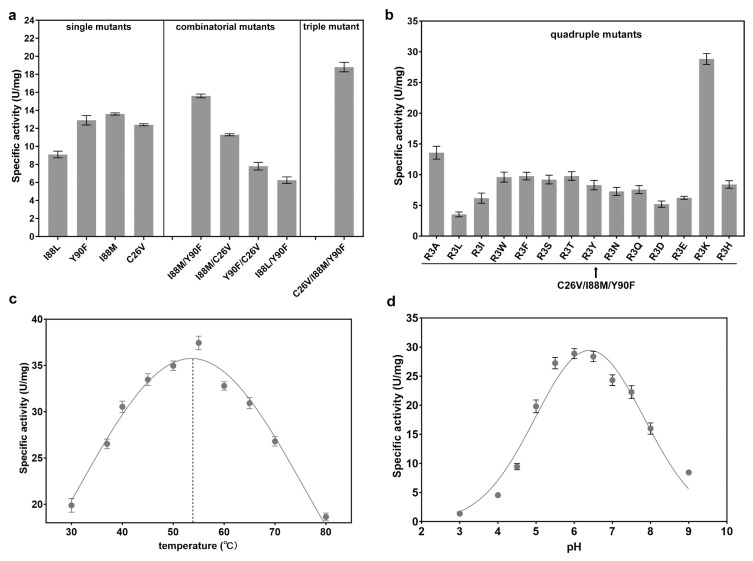
Combinatorial mutagenesis of CjADC and characterization of the mutants. (**a**) Analysis and comparison of the specific activities of the single mutants, double mutants, and triple mutants. (**b**) Determination of the specific activities of the quadruple mutants using C26V/I88M/Y90F triple mutant as a template. (**c**) Determination and characterization of the optimal temperature of the quadruple mutant C26V/I88M/Y90F/R3K. (**d**) Determination of the optimal pH of the quadruple mutant C26V/I88M/Y90F/R3K. All the experiments were independently repeated in triplicate. The data are shown by as ± s.d.

To characterize the specific activity and other enzymatic properties, the four mutants were overexpressed and purified. Three mutants, Y90F, I88M, and C26V, exhibited higher specific activity than the wild-type CjADC. The specific activity of I88M was 13.6 U/mg (1.2 times higher than that of the wild type), which was the highest among them (Figure 5a). Therefore, in this round of engineering, three evolved mutants were successfully obtained through single mutation.

### 3.5. Generation of the Multiple-Site Mutations, Screening of the Mutants, and Characterization of the Catalytic Properties

Based on the first round of evolution, we conducted the next round of engineering through combinatorial mutagenesis. We first created several double mutants using pairwise combinations. Then, their specific activities were determined. As shown in Table 3, among the four double mutants, I88M/Y90F showed the highest specific activity (15.8 U/mg), which was approximately 1.5 times higher than that of the wild type. Additionally, the Km of I88M/Y90F was reduced by about 1.2 times, while the catalytic efficiency (kcat/Km) increased by 1.43 times compared to the wild-type enzyme. However, the other three double mutants displayed decreased specific activities, catalytic efficiency, and increased Km, suggesting that certain combinations of positive mutations can impair catalytic activity. This is a universal effect observed in the evolution of many other enzymes, resulting in mutational epistasis in protein evolution [38].

Therefore, considering the epistatic effect, we performed a third round of evolution by combining I88M/Y90F with C26V. Interestingly, the specific activity of the C26V/I88M/Y90F mutant increased to 18.8 U/mg. Notably, the Km of this mutant was lower than that of the wild-type enzyme, I88M/Y90F, and C26V. Additionally, the catalytic efficiency significantly increased to 61.2 mM^−1^·s^−1^, more than two times that of the wild-type CjADC (29.7 mM^−1^·s^−1^) (Table 3).

Previous studies have shown that R3 residue might be involved in the self-processing of the enzyme from *C. glutamicum*, although it is far from the residues around the active center [33]. However, the contribution of R3 to catalytic activity has remained unknown. Here, to investigate the effect of R3 on CjADC evolution, a fourth round of mutagenesis was carried out by mutating R3 of C26V/I88M/Y90F mutant to 14 different amino acids, generating an array of quadruple mutants. The specific activity of C26V/I88M/Y90F/R3K was 28.8 U/mg, which was the highest among the 14 mutants. Compared to the wild-type CjADC and C26V/I88M/Y90F, the specific activity increased by 2.7 times and 1.5 times, respectively. In contrast, C26V/I88M/Y90F combined with other R3 mutations showed decreased specific activity, indicating an epistatic effect of this residue during protein evolution. We characterized the kinetic parameters of the quadruple mutant and compared them with those of the double and triple mutants. The data revealed that the Km of C26V/I88M/Y90F/R3K was 2.2 mM, significantly lower than that of the wild-type enzyme and other mutants. Furthermore, the catalytic efficiency of the mutant increased by 3.2 times compared to the wild type. Additionally, the combination of R3K with C26V/I88M/Y90F led to an increase in catalytic efficiency by 1.5 times. These results suggest that mutations targeting medium conserved residues around the active center promote substrate binding and subsequently elevate enzymatic activity (Table 3).

We further investigated two parameters for the quadruple mutant, the optimal temperature and the optimal pH, and found that they were 52.5 °C and 6.4, respectively. These two parameters were equivalent to those of the wild type, indicating that the engineering of CjADC specifically evolved towards enhancing the catalytic efficiency.

### 3.6. Identification and Comparison of the Thermostability and pH Stability

**Figure 6:**
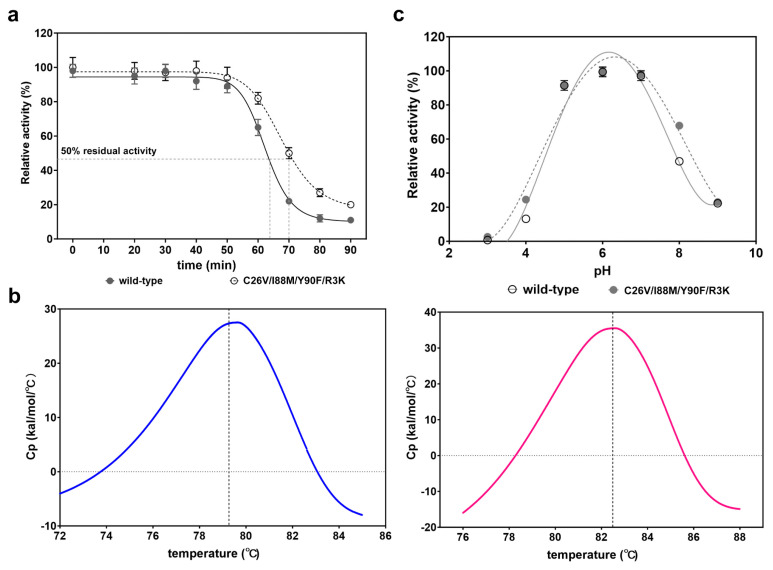
Characterization and evaluation of thermostability and pH stability of the quadruple mutant. (**a**) Determination and comparison of the thermostability of the wild-type CjADC and the mutant C26V/I88M/Y90F/R3K. t1/2, which is the time at which the enzyme can retain 50% activity after heating treatment, was used to evaluate the thermostability. The purified C26V/I88M/Y90F/R3K was incubated at 70 °C for different times, and then the retained activity was determined and is represented as a percentage compared to that without heating treatment. (**b**) Tm values of the wild-type CjADC (left) and the mutant C26V/I88M/Y90F/R3K (right) were determined using Nano Differential Scanning Calorimetry (Nano DSC). The gray dashed lines indicate the Tm of the two enzymes. (**c**) Determination and comparison of the pH stability of the wild type and the mutant C26V/I88M/Y90F/R3K. The wild-type enzyme and the mutant were firstly in the buffer of different pH. Then, the residual activities of the enzymes were determined, and the data are shown as a percentage compared to that without treatment. All the experiments were independently performed in triplicate, and the data are shown as mean ± s.d.

As the optimal temperature and pH of the quadruple mutant were not significantly different from those of the wild type, our next step was to investigate whether other properties had changed. Therefore, we conducted tests to assess the thermostability and pH stability of the quadruple mutant. After 70 °C incubation for different times from 0 to 90 min, we observed that the residual activity of the C26V/I88M/Y90F/R3K remained at 95% within 40 min treatment, similar to that of the wild-type enzyme. However, the relative activity began to decrease after 50 min of treatment. After 70 min incubation, the wild-type enzyme only retained 20% of its activity, while the quadruple mutant still maintained about 55% of activity (Figure 6a). t1/2 is a parameter for evaluating thermostability, which signifies the time when the enzyme can retain 50% of its residual activity after heat treatment. Here, we observed that the t1/2 of the quadruple mutant was 70.1 min, which was longer than that of the wild-type enzyme (62.8 min). To further confirm the results, we introduced another parameter, the protein melting point ™, to quantify the property. As expected, the Tm of C26V/I88M/Y90F/R3K was 82.5 °C (Figure 6b, right), marginally higher than 79.6 °C of the wild-type enzyme (Figure 6b, left). These findings indicate that the mutations of these residues also enhance thermostability to a certain extent.

Furthermore, the pH stability between the wild type and the mutant was also determined and compared. The results showed that they had nearly equivalent pH stability in the range of pH 3 to pH 9 (Figure 6c).

### 3.7. Insight into the Mechanism of Catalytic Activity Enhancement through Structural Analysis

**Figure 7:**
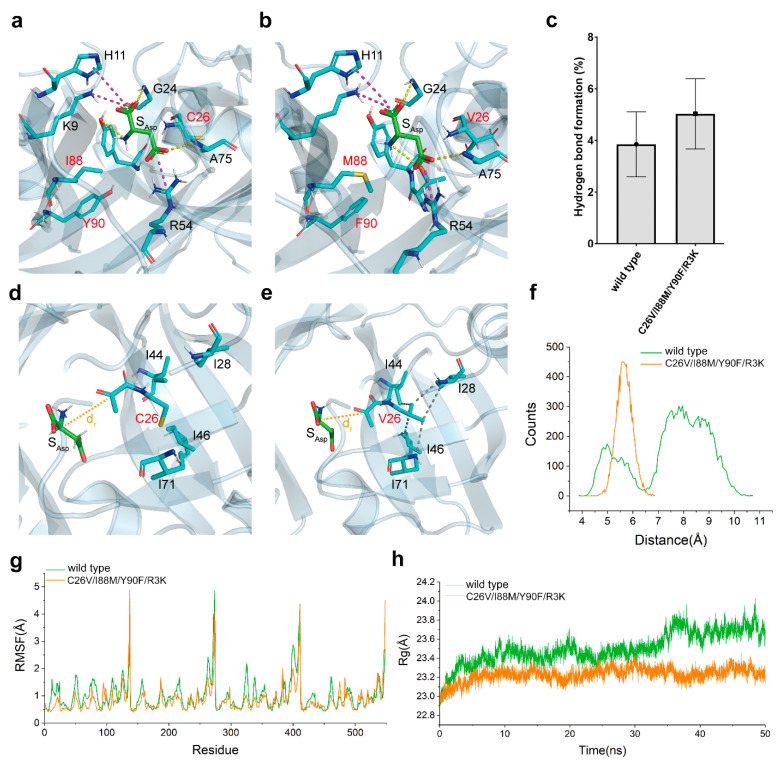
The proposed mechanism of evolved catalytic properties. A close-up view of the catalytic pocket of the wild-type CjADC (**a**) and the mutant (**b**) docked with L-aspartate. The residues around the substrate are shown as sticks. These residues to be mutated in (**a**,**b**) are shown in red. The dashed lines denote the potential interactions of L-aspartate with their relevant residues. The yellow and magenta dashed lines indicate hydrogen bonds and salt bridges, respectively. (**c**) Hydrogen bond occupancy for the wild-type CjADC and the mutant. The close-up view of the reaction distance (dr) distribution between the active site and the Cα of L-aspartate in wild-type CjADC (**d**) and C26V mutant (**e**). (**f**) Comparison of the average distance between L-aspartate and the active site of wild-type CjADC (wild type) and the mutant (C26V/I88M/Y90F/R3K). (**g**) Analysis of RMSF based on 50-ns MD simulation. (**h**) The analysis for radius of gyration (Rg) of wild-type CjADC (wild type) and the mutant (C26V/I88M/Y90F/R3K) based on 50 ns MD simulation.

The structural model of both wild-type CjADC and C26V/I88M/Y90F/R3K were created. L-aspartate was then docked into the catalytic pocket of each model. After optimizing the structure of enzyme–substrate complexes using the relax module of Rosetta software, the interactions between L-aspartate and CjADC were analyzed. As shown in Figure 7a,b, the mutations I88M and Y90F were situated in the catalytic pocket, and L-aspartate bound to both wild-type CjADC and the mutant in a similar pattern. Within the model, α-carboxyl group of L-aspartate formed salt bridges with K9 and H11, while the β-carboxyl group was bound by R54. Hydrogen bonds were formed between the amino group of the protein backbone of G24 and A75 with L-aspartate, and between the amino group of L-aspartate and T57 or Y58. To understand the dynamics of the interaction between L-aspartate and CjADC, a 50 ns conventional molecular dynamics (cMD) simulation was conducted (Supplementary Figure S3). The occupancy of hydrogen bonds formed by key residues was statistically analyzed. Compared to wild-type CjADC, the hydrogen bonds formed between the mutant and L-aspartate were found to be more stable than those formed in the wild type (Figure 7c).

Notably, the side chain of C26 was oriented toward a hydrophobic pocket, which was mainly composed of several hydrophobic residues I28, I44, I46, L55, A59, I69, I71, A75, V85, and I87 within a 5 Å distance from C26 (Figure 7d). By introducing a C26V mutation, suitable hydrophobic interactions with I28, I44, I46, and I71 were newly created (Figure 7e). These hydrophobic interactions likely contributed to the stabilization of the active site, allowing the pyruvoyl group in the active site readily react with L-aspartate. To confirm this speculation, the reaction distance (dr) distribution between the active site and the Cα of L-aspartate was calculated. According to Figure 7d,e, the dr in the mutant significantly differed from that in the wild-type enzyme (Figure 7f). Generally, close distances of dr (4–6 Å) favor the reaction, while far distances (7–10 Å) are unfavorable. In the mutant, the dr remained constantly at a low level (5–6.5 Å) throughout the MD simulation.

RMSF analysis demonstrated that these mutations greatly reduced the structural flexibility, thereby promoting the stability of the mutant I88M/Y90F/ C26V/ R3K (Figure 7g). In addition, the radius of gyration (Rg), which is commonly used to evaluate the thermostability and structural intensity of multimeric proteins, was calculated from the MD simulation. The lower Rg of the mutant indicated a higher stable octamer structure compared to the wild-type CjADC (Figure 7h).

Thus, these results show that the introduced mutations increased the structural stability of the mutant by promoting the frequency of hydrogen bond formation and creating a more hydrophobic region around the active center, thereby facilitating the binding of L-aspartate to the active center and stabilizing the substrate orientation.

### 3.8. Scale-Up of the Recombinant CjADC-Producing Strains and Identification of the Bioconversion Efficiency

As a pivotal enzyme, ADCs from *C. glutamicum* and *B. subtilis* have been extensively used in the bioconversion of L-aspartate to β-alanine in the metabolism of D-pantothenic acid. However, the main constraint in achieving high product titer is the low activity of these enzymes [4,36]. Based on these results, we confirmed that the quadruple mutant and CjADC are superior to the currently utilized enzymes from the two sources.

**Figure 8:**
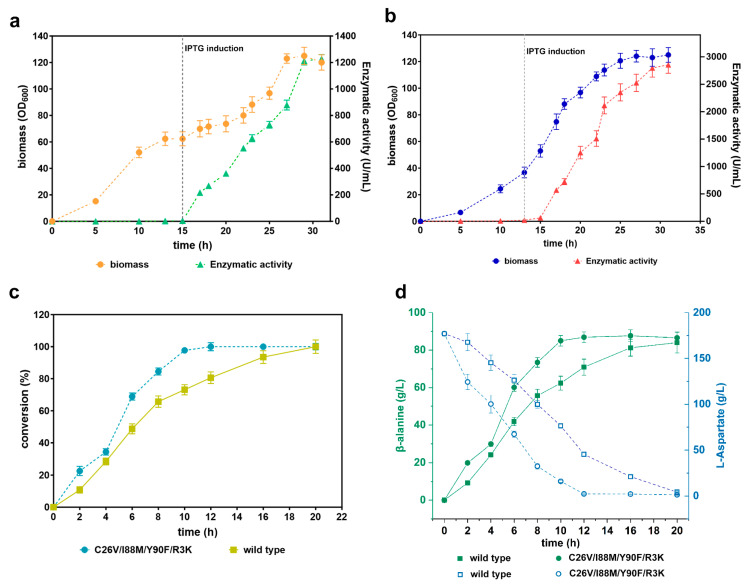
Validation of the efficiency of whole-cell bioconversion using the mutant C26V/I88M/Y90F/R3K. (**a**) Scale-up fermentation of *E. coli* (BL21) cells producing wild-type CjADC in a 5 L bioreactor. The left y-axis represents the biomass, while the right y-axis represents the level of produced enzyme. The gray dashed vertical line indicates the induction time with IPTG. (**b**) Scale-up fermentation of *E. coli* (BL21) cells producing C26V/I88M/Y90F/R3K in a 5 L bioreactor, presented in a similar manner to (**a**). (**c**) Determination of β-alanine conversion efficiency from L-aspartate using the wild-type CjADC and C26V/I88M/Y90F/R3K through a whole-cell bioconversion system. (**d**) Analysis and comparison of β-alanine production and substrate depletion during the bioconversion process using wild-type CjADC and C26V/I88M/Y90F/R3K through a whole-cell bioconversion system. All the experiments were independently repeated in triplicate. The data are shown as mean ± s.d.

To evaluate the application of C26V/I88M/Y90F/R3K in bioconversion, we performed an investigation of whole-cell bioconversion. We first scaled up the recombinant *E. coli* BL21(DE3) cells producing both the wild-type CjADC and C26V/I88M/Y90F/R3K in a 5 L bioreactor. The levels of the produced enzymes were monitored by assessing their enzymatic activity throughout the fermentation process. The data revealed that the biomass of the two recombinant strains reached a cell density (OD600) of over 120. Concurrently, the enzymatic activity exhibited a gradual increase during the fermentation process after induction. After 31 h of fermentation, the wild-type enzyme achieved the highest enzymatic activity of 1227.82 U/mL, whereas the mutant enzyme reached an even higher level of 2853.87 U/mL (Figure 8a,b).

Then, we performed whole-cell transformation using these fermented cells. Firstly, the cells were collected, and their cell density (OD600) was adjusted to 40 in a phosphate saline buffer (pH 7.0). Next, we added 1 M of substrate to the cell suspension to initiate the transformation process. Samples were periodically collected and subjected to HPLC. We observed that the conversion rate of the cells containing the mutant C26V/I88M/Y90F/R3K and the wild-type enzyme gradually increased over time. The highest conversion rate for the mutant was reached at 12 h, while the wild type achieved it at 20 h, indicating that the mutant’s conversion rate is faster than that of the wild type (Figure 8c).

Subsequently, we assessed the bioconversion yield of the two enzymes. The results showed that the yield of β-alanine via the quadruple mutant increased over time until it reached a peak at 12 h, with a final product yield of 88.6 g/L. Notably, the system was able to transform ≈50% of the substrate within 4 h. In contrast, the system with the wild type produced an equivalent yield of β-alanine but required much more time (20 h) to achieve the highest yield level. As the enzymatic activity of CjADC and the mutant was not performed with high concentrations of L-aspartate over 1 M (Supplementary Figure S4), the bioconversion of L-aspartate over 1 M was not performed in this study.

## 4. Conclusions and Perspective

In this study, we discovered a novel L-aspartate α-decarboxylase from *Corynebacterium jeikeium* that has higher enzymatic activity than previously reported enzymes and successfully improved the enzymatic performance through a structure-guided semi-rational design. Whole-cell bioconversion revealed that the variant C26V/I88M/Y90F/R3V can efficiently convert L-aspartate to β-alanine. These results provide a superior biocatalyst for the overproduction of β-alanine and the derivatives for biocatalysis and metabolic engineering.

## Figures and Tables

**Table 1:** Enzymatic parameters of ADCs from different sources.

	CorJei	BacSub	CorGlu	LisMon	BacThe
Specific activity (U/mg)	10.7 ± 0.3	7.2 ± 0.2	6.3 ± 0.1	5.8 ± 0.1	2.6 ± 0.2
*K*_m_ (mM)	3.6 ± 0.2	4.2 ± 0.3	4.5 ± 0.1	6.0 ± 0.2	8.9 ± 0.4
*k*_cat_ (s^−1^)	106.8 ± 0.2	90.6 ± 0.3	80.8 ± 0.2	69.6 ± 1.1	52.5 ± 1.2
*k*_cat_/*K*_m_ (mM^−1^·s^−1^)	29.7 ± 0.7	21.6 ± 0.6	17.8 ± 0.4	11.6 ± 0.3	5.9 ± 0.2

CorJei stands for the ADC from *Corynebacterium jeikeium*; BacSub stands for ADC from *Bacillus;* CorGlu stands for ADC from *Corynebacterium glutamicum*; LisMon stands for ADC from *Listeria Monocytogenes*; BacThe stands for ADC from *Bacteroides thetaiotaomicron.*

**Table 2:** The specific activity of the screened mutants.

No.	Mutants	Specific Activity (U/mg)
M10	I88L	9.1 ± 0.2
M11	Y90F	12.9 ± 0.2
M12	C26V	12.4 ± 0.1
M23	I88M	13.6 ± 0.3

**Table 3:** Kinetic parameters of the CjADC mutants.

	I88M/Y90F	I88M/C26V	Y90F/C26V	I88L/Y90F	C26V/I88M/Y90F	I88M/Y90F/C26V/R3K
Specific activity (U/mg)	15.8 ± 0.2	11.3 ± 0.1	7.8 ± 0.4	6.2 ± 0.3	18.8 ± 0.5	28.8 ± 0.8
*K*_m_ (mM)	3.2 ± 0.4	3.6 ± 0.2	4.1 ± 0.3	3.9 ± 0.1	2.6 ± 0.6	2.2 ± 0.5
*k*_cat_ (s^−1^)	136.8 ± 0.3	110.5 ± 1.3	98.6 ± 1.2	88.6 ± 2.8	171.5 ± 0.8	209.2 ± 0.8
*k*_cat_/*K*_m_ (mM^−1^·s^−1^)	42.7 ± 0.7	30.5 ± 0.4	24.0 ± 0.3	22.7 ± 0.5	61.2 ± 1.3	95.1 ± 1.6

## Data Availability

Data is contained within the article or Supplementary Materials.

## Supplementary Materials

The following supporting information can be downloaded at: https://www.mdpi.com/article/10.3390/foods12244423/s1, Table S1: Strains and plasmids, Table S2: Primers used in this study, and Table S3: Binding free energy for virtual mutagenesis; Figure S1: SDS-PAGE analysis of recombinant expression of PanD in *Escherichia coli*, Figure S2: Modeling and checking of L-aspartate α-decarboxylase from *Corynebacterium jeikeium*, Figure S3: Fifty nanosecond conventional molecular dynamic simulation of the Wild-type CjADC (WT) and the quaternary mutant (I88M/Y90F/R3K/C26V), and Figure S4: Enzymatic inhibition by high concentrations of L-aspartate.
