# Supplementary material for: Discovery and Engineering of a Novel Bacterial L-Aspartate α-Decarboxylase for Efficient Bioconversion
Source: Foods. 2023 Dec 10;12(24):4423. doi: 10.3390/foods12244423 (PMC10743139; doi:10.3390/foods12244423)
Supplement: Supplementary file 1 [file foods-12-04423-s001.zip › foods-2737018-supplementary.pdf]

Discovery and Engineering of a Novel Bacterial L-Aspartate α-Decarboxylase for Efficient Bioconversion

Wenjing Cui, Hao Liu, Yan Ye, Laichuang Han*, Zhemin Zhou

School of Biotechnology, Jiangnan University, Wuxi Jiangsu 214122

Corresponding author: Laichuang Han

Email: hanlaichuang@jiangnan.edu.cn

Supplementary Tables

Table S1. Strains and plasmids

Strains and plasmids	introduction	source
E.coil JM109	cloning host	Lab stock
E.coil BL21(DE3)	Expressing host	Lab stock
pET-24a(+)plasmid vector	Kana resistance、His-tag	Lab stock
pET-28a(+)plasmid vector	Kana resistance、His-tag	Lab stock
pET-28a-CorGlu	Kana resistance、C-terminal His-tag	Lab stock
pET-28a-BacSub	Kana resistance、C-terminal His-tag	This study
pET-28a-CorJei	Kana resistance、C-terminal His-tag	This study
pET-28a-LisMon	Kana resistance、C-terminal His-tag	This study
pET-28a-StaAur	Kana resistance、C-terminal His-tag	This study
pET-28a-BacThe	Kana resistance、C-terminal His-tag	This study
pET-28a-CorJei	Kana resistance、strep-tag	This study
pET-28a-CorJei-I88M-Y90F	Kana resistance、C-terminal His-tag	This study

pET-28a-CorJei-I88M-Y90F-C26V	Kana resistance、C-terminal His-tag	This study
pET-28a-CorJei-I88M-Y90F-R3K	Kana resistance、C-terminal His-tag	This study

Table S2. Primers used in this study

Primer name	Primer sequences（5'→3'）
pET28a-V-F	GGTATATCTCCTTCTTAAAGTTAAACAAAATTATTTCTAGAG
pET28a-V-R	CACCACCACCACCACCACTGAG
CorGlu-i-F	CTTTAAGAAGGAGATATACCATGCTGCGTACCATCCTG
CorGlu-i-R	GTTAGCAGCCGGATCTCAGTGGTGGTGGTGGTGGTGAATACTACG
BacSub-i-F	CTCAGTGGTGGTGGTGGTGGTGCAAAATTGTACGGGCTGGTTCG
BacSub-i-R	GTTTAACTTTAAGAAGGAGATATACCATGTATCGAACAATGATGA
CorJei-i-F	GTTAGCAGCCGGATCTCAGTGGTGGTGGTGGTGGTGCGCTTCCG
CorJei-i-R	TTAACTTTAAGAAGGAGATATACCATGCTGCGCACCATGCTGAAA
LisMon-i-F	ATCTCAGTGGTGGTGGTGGTGGTGCAGGGTGGTATGCGCTTTTTC
LisMon-i-R	ACTTTAAGAAGGAGATATACCATGTTTCGCACCATGATGAACGGC
BacThe-i-F	TCAGTGGTGGTGGTGGTGGTGCTTCACTACGCTATTCGTCGCAG
BacThe-i-R	GTTTAACTTTAAGAAGGAGATATACCATGATGATTGAAGTGTTGA

StaAur-i-F	TCAGTGGTGGTGGTGGTGGTGTAACACTATCGTATTTTCTTTTTCA
StaAur-i-R	GTTTAACTTTAAGAAGGAGATATACCATGATGAATGCTAAAATTCA

Table S3. Binding free energy for virtual mutagenesis

Residue	Wild type	mutation	ddG(kcal/mol)
90	Tyr	Trp	-8.296
88	Ile	Leu	-6.85
90	Tyr	Phe	-6.286
88	Ile	Met	-5.801
88	Ile	Val	-5.023
26	Cys	Leu	-4.946
88	Ile	Tyr	-3.953
88	Ile	Glu	-3.951
26	Cys	Phe	-3.726
26	Cys	Ile	-3.595
11	His	Val	-3.572
72	Asn	Leu	-3.458
72	Asn	Tyr	-3.137
55	Leu	Phe	-2.94
72	Asn	Val	-2.332
11	His	Ile	-2.325

56	Thr	Trp	-2.283
56	Thr	Ile	-2.121
72	Asn	Met	-1.87
56	Thr	Val	-1.762
49	Ile	Leu	-1.518

Supplementary Figures

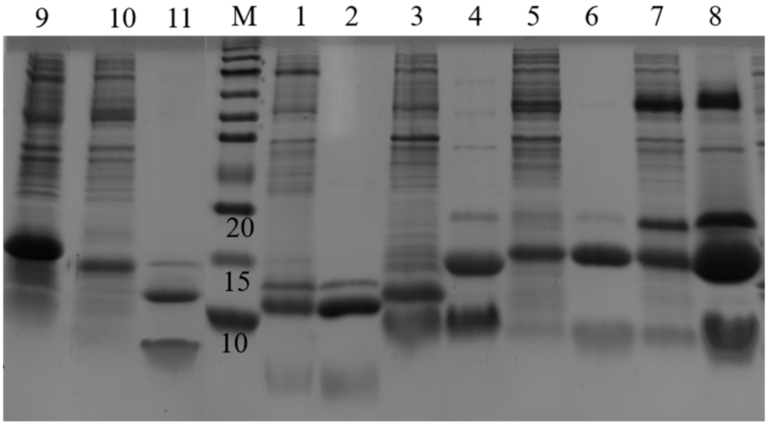

Figure S1. SDS-PAGE analysis of recombinant expression of PanD in *Escherichia coli*

1：BasThe whole-cell biotransformation system (WCB)；2：BasThe purified enzyme；3：CorJei WCB system；4： CorJei purified enzyme；5：BacSub WCB system；6：BacSub purified enzyme；7：LisMon WCB system； 8：LisMon purified enzyme；9：StaAur insoluble precipitation；10：CorGlu WCB system；11：CorGlu purified enzyme；M：Marker

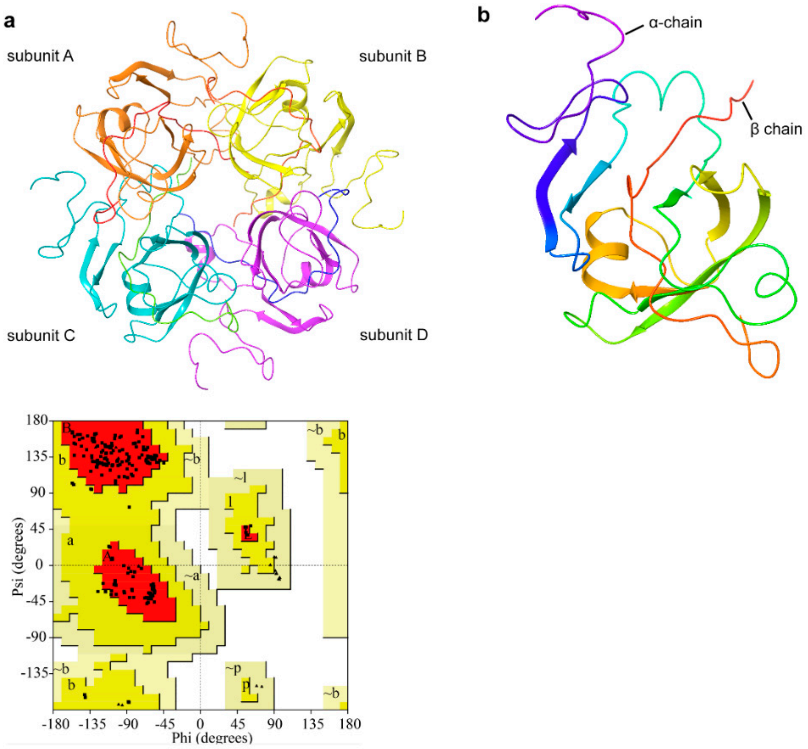

Figure S2. Modeling and checking of L-aspartate α-decarboxylase from Corynebacterium jeikeium.

(a) the assembled quaternary structure of ADC from Corynebacterium jeikeium. (b) The Ramachandran Diagram showing the model.

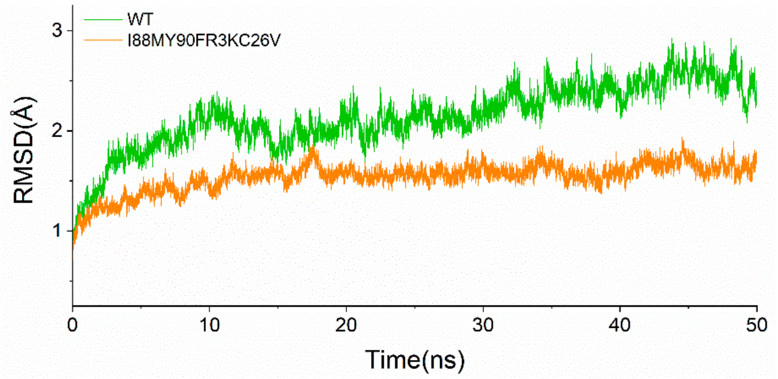

Figure S3. Fifty nanosecond conventional molecular dynamic simulation of the Wild-type CjADC (WT) and the quaternary mutant (I88M/Y90F/R3K/C26V).

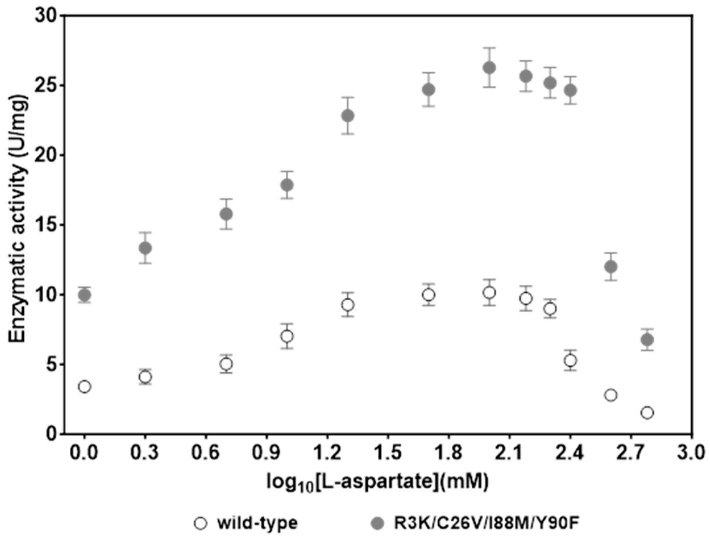

Figure S4. Enzymatic inhibition by high concentrations of L-aspartate
